# Assessment of Extracellular Matrix Fibrous Elements in Male Dermal Aging: A Ten-Year Follow-Up Preliminary Case Study

**DOI:** 10.3390/biology13080636

**Published:** 2024-08-20

**Authors:** Bogusław Machaliński, Dorota Oszutowska-Mazurek, Przemyslaw Mazurek, Mirosław Parafiniuk, Paweł Szumilas, Alicja Zawiślak, Małgorzata Zaremba, Iwona Stecewicz, Piotr Zawodny, Barbara Wiszniewska

**Affiliations:** 1 Department of General Pathology, Pomeranian Medical University in Szczecin, 70-111 Szczecin, Poland; malgorzata.zaremba@wum.edu.pl (M.Z.); iwona.stecewicz@pum.edu.pl (I.S.); drpiotrzawodny@wp.pl (P.Z.); 2 Department of Histology and Embryology, Pomeranian Medical University in Szczecin, 70-111 Szczecin, Poland; dorota.oszutowska.mazurek@pum.edu.pl (D.O.-M.); barbara.wiszniewska@pum.edu.pl (B.W.); 3 Department of Signal Processing and Multimedia Engineering, West Pomeranian University of Technology in Szczecin, 70-310 Szczecin, Poland; 4 Department of Forensic Medicine, Pomeranian Medical University in Szczecin, 70-111 Szczecin, Poland; miroslaw.parafiniuk@pum.edu.pl; 5 Department of Social Medicine and Public Health, Pomeranian Medical University in Szczecin, 71-210 Szczecin, Poland; pawel.szumilas@pum.edu.pl; 6 Department of Interdisciplinary Dentistry, Pomeranian Medical University in Szczecin, 70-111 Szczecin, Poland; alicja.zawislak@pum.edu.pl; 7 Department of Experimental and Clinical Pharmacology, Centre for Preclinical Research (CBP), Medical University of Warsaw, 02-097 Warsaw, Poland

**Keywords:** aging skin, male skin, 10-year interval, dermis, type I and III collagen, elastin, generalized Pareto distribution analysis, image analysis

## Abstract

**Simple Summary:**

This study aimed to examine the aging process of fibrous elements in the dermis of male volunteers over a 10-year period (47–57 years). It was observed that the aging process included visible changes in the content and organization of type I collagen fibers, resulting in changes in fibroblast morphology, and changes in type III collagen and elastic fibers. For the first time, this study presents a comparison of the dynamics of changes in morphology of the dermis in the same volunteer over a time interval of 10 years.

**Abstract:**

Skin aging is a complex phenomenon influenced by multiple internal and external factors that can lead to significant changes in skin structure, particularly the degradation of key extracellular matrix (ECM) components such as collagen and elastic fibers in the dermis. In this study, we aimed to meticulously assess the morphological changes within these critical fibrous ECM elements in the dermis of the same volunteer at age 47 and 10 years later (2012 to 2022). Using advanced histological staining techniques, we examined the distribution and characteristics of ECM components, including type I collagen, type III collagen, and elastic fibers. Morphological analysis, facilitated by hematoxylin and eosin staining, allowed for an accurate assessment of fiber bundle thickness and a quantification of collagen and elastic fiber areas. In addition, we used the generalized Pareto distribution for histogram modeling to refine our statistical analyses. This research represents a pioneering effort to examine changes in ECM fiber material, specifically within the male dermis over a decade-long period. Our findings reveal substantial changes in the organization of type I collagen within the ECM, providing insight into the dynamic processes underlying skin aging.

## 1. Introduction

Aging is a natural process that affects all body organs, resulting in gradual changes in their structure and function. The most noticeable is the aging of the skin, the outermost layer of the body [1]. Resulting from the interaction of biological, biochemical, and physical factors, skin aging is a multifaceted phenomenon associated with physiological changes induced by a combination of endogenous and exogenous factors [2]. Internal factors contribute to natural, chronological, spontaneous, and biological changes in the appearance and function of the skin, which can be exacerbated by external factors [3]. This interaction of both types of factors can lead to morphological and physiological changes in the skin, especially in skin areas that are exposed to sunlight [4].

The human skin consists of two primary layers: the epidermis and the dermis. The epidermis is the superficial epithelium, while the dermis is adjacent to the epidermis and makes up most of the skin. Immediately below the dermis is a layer of adipose tissue associated with loose connective tissue that forms the hypodermis or subcutaneous fascia, which connects the dermis to the underlying fascia (fibrous tissue) of the bones and muscles [5]. The epidermis consists of a stratified keratinized squamous epithelium with keratinocytes as the primary cell population resting on the basement membrane [6,7]. The dermis is composed of two layers of connective tissue—the papillary or superficial layer and the reticular or deeper layer [8]—each with a different extracellular matrix (ECM) content and profile. The base of the ECM is an amorphous gel-like material composed of glycosaminoglycans, proteoglycans, glycoproteins, and embedded fibers. The ECM contains proteins that form intricate networks contributing to tissue characteristics such as stiffness, elasticity, and resilience [9,10].

The papillary layer is composed of loose connective tissue with a fine collagen network consisting primarily of type I and type III collagens, elements of the elastic fiber system, and loosely organized ECM elements with a higher cellular content. In addition, the elastic fiber system includes very thin oxytalan fibers composed of fibrillin-rich microfibrils. Elaunin fibers, on the other hand, contain a network of fibrillin microfibrils interspersed with elastin [11,12]. Mature elastic fibers consist of cross-linked elastin molecules in a core and a mantle of fibrillin microfibrils, mainly fibrillin-1. The reticular layer is thicker and consists of connective tissue with a dense network of collagen and elastic fibers [13]. It is less cellular than the papillary layer [14].

The ECM of the human skin is composed primarily of two types of collagen fibers: type I collagen (approximately 90%) and type III collagen (approximately 10%). In addition, mature elastic fibers are present in the fibrous component of the ECM. Type I collagen fibers form thick, densely packed bundles running parallel to the skin surface. Thicker than those in the papillary dermis, elastic fibers interweave around collagen fibers and are more abundant near sweat and sebaceous glands [10,13]. The two layers are primarily composed of dermal fibroblasts, which are resident cells responsible for synthesizing and degrading the ECM. The ECM plays a critical role in maintaining tissue homeostasis [15,16] by regulating the proliferation and migration of these cells. Cell surface receptors known as integrins facilitate fibroblast attachment to collagen fibrils, with the integrin β1-subunit playing a critical role in mechanosignaling [17,18].

As individuals age, the skin undergoes changes, particularly in the dermal ECM components [19], due to a decline in morphological and physiological functions. It has been observed that the number of fibroblasts decreases, leading to reduced synthesis of ECM components such as collagen and elastin. Collagen fibers undergo significant changes during aging, including both quantitative and structural changes [20,21]. In addition, collagen bundles become thinner, the ratio of type I to type III collagen is altered, and elastic fibers show fragmentation and degeneration [3,22]. These morphological features associated with changes in the dermal ECM are attributed to the increased activity of matrix-degrading enzymes and matrix metalloproteinases produced by fibroblasts. Both intrinsic and extrinsic factors play a role in these processes.

It has been postulated that air pollution may contribute to the skin aging process [2]. Recent research indicates that air pollution is a significant environmental health risk factor, potentially harmful to the skin [23,24]. The rate of skin aging can also be significantly affected by exposure to ultraviolet light. Photoaging interacts with physiological aging, accelerating the process, with chronic sun exposure believed to account for up to 90% of visible skin aging [25]. This exposure leads to the degradation of elastic fibers and their accumulation as solar elastosis, whereas in normal aging, fibers in the dermis form abnormal clumps. In addition, oxytalan fibers degrade and are often absent from the skin [26,27].

The structures of female and male skin are similar; however, sex significantly influences facial anatomy and behavior, which are key factors in the aging process. Male skin is approximately 25% thicker, and characteristics such as hydration, pH, pigmentation, and hormonal regulation differ from those of female skin. In addition, adult male skin has a significantly higher collagen content compared with adult female skin [28,29]. Research on facial skin aging has also revealed differences in pigmentation and hormonal regulation between males and females.

Differences in skin structure based on race and ethnicity can influence the manifestation and signs of facial aging. A study of Chinese subjects found a marked and almost linear progression of aging with age in both sexes, except for the lip area, where aging progressed faster and was more pronounced in women [28]. In contrast, research on Caucasian French subjects showed only a slight difference in facial skin aging between sexes, with the lower part of the face playing an important role in age assessment [29]. Overall, studies suggest that the quality of dermal extracellular matrix (ECM) components tends to deteriorate earlier in women than in men, with hormonal changes, sun exposure, and lifestyle contributing to this difference [21].

Our study aimed to evaluate the morphological changes of fibrous ECM elements in the dermis of the same subject at the ages of 47 and 57 years (2012–2022).

## 2. Materials and Methods

### 2.1. Materials

The study material consists of human skin samples from a male volunteer of European descent with normal white skin. The volunteer was 47 years old in 2012 (archival material [30]) and 57 years old in 2022. The skin sections were obtained from the postauricular area of a volunteer who lives in a temperate climate, does not smoke cigarettes or drink strong alcohol, consumes high-quality food, is healthy, and has not undergone any surgical procedures. He is a medical doctor, is actively involved in sports, has short hair, and has not had an occupation that required prolonged exposure to the outdoors. The area from which the samples were taken had limited sun exposure. The decision on where to harvest the tissue was also based on aesthetic considerations important to the patient. While external factors such as air pollution cannot be completely ruled out, we believe that internal factors were the main cause of the changes in the dermis in this case.

### 2.2. Histological Examination of Skin

Two samples were obtained from the postauricular area by punch biopsy (5 mm diameter) according to the Declaration of Helsinki and with the approval of the local ethics committee of Pomeranian Medical University. The tissues were fixed in 4% paraformaldehyde for at least 24 h, followed by a series of washes under sterile conditions with absolute ethanol (3 times for 3 h), absolute ethanol with xylene (1:1) (2 times for 1 h), and xylene (3 times for 20 min). After saturation in liquid paraffin for 3 h, the samples were embedded in liquid paraffin. Subsequently, 3–5 μm serial sections were cut with a microtome (Microm HM340E Thermo Fisher Scientific, Walldorf, Germany) and mounted on polysine microscope slides (Thermo Scientific, Leicestershire, UK; cat. no. J2800AMNZ; Thermo Fisher Scientific, Braunschweig, Germany). The sections were then deparaffinized in xylene, rehydrated in decreasing concentrations of ethanol, and finally used for histological staining.

For each histological staining method, two slides were prepared, each containing at least five serial sections. Tissue morphology was evaluated by standard staining techniques using hematoxylin (Mayer’s hematoxylin, REF. 98.099.4 STAMAR, Dąbrowa Górnicza, Poland) and eosin (Eosin Y solution 2%, REF. 98.118.3 STAMAR, Dąbrowa Górnicza, Poland). Collagen fibers were visualized using Mallory’s trichrome method, which involves staining with three different dyes—carbol fuchsin for nuclear staining, orange G for cytoplasm, and aniline blue for selective collagen staining—resulting in a deep blue color (Bio-Optica, Milano, Italy, cat. no. 04-020802).

The silver impregnation method is commonly utilized to visualize type III collagen, specifically reticular fibers. This method involves two steps: initial impregnation with iron salt and the use of an unstable diamine complex (ammonia solution) as a source of silver. As a result of this process, the fibers appear black (Bio-Optica, Milano, Italy, cat. no. 04-020802).

Two methods were employed to identify mature elastic fibers. Weigert’s method involves a reaction between resorcin and basic fuchsin in the presence of ferric chloride (Bio-Optica Milano, Italy, cat. no. 04-052812) [30]. Weigert’s method and orcein staining are two commonly used staining techniques in histology. Orcein staining, on the other hand, requires the use of several reagents, such as potassium permanganate solution, acid activation buffer, oxalic acid solution, alcoholic reagent for incubation, orcein solution according to Shikata, and differentiation solution. It should be mentioned that the fibrillin-rich microfibrils, including oxytalan fibers and elaunin fibers, are not visible with the staining used in this study.

All histological stainings were performed under the manufacturer’s protocols. Following this, Entellan^TM^ new (a rapid mounting medium for microscopy, Sigma-Aldrich, Darmstadt, Germany, ref. 1.07961) was applied to all sections on the microscope slides, which were then covered with coverslips. The slides were analyzed using a microscope (Leica DM5000B, Wetzlar, Germany).

### 2.3. Digital Image Acquisition and Segmentation

**Figure 1:**
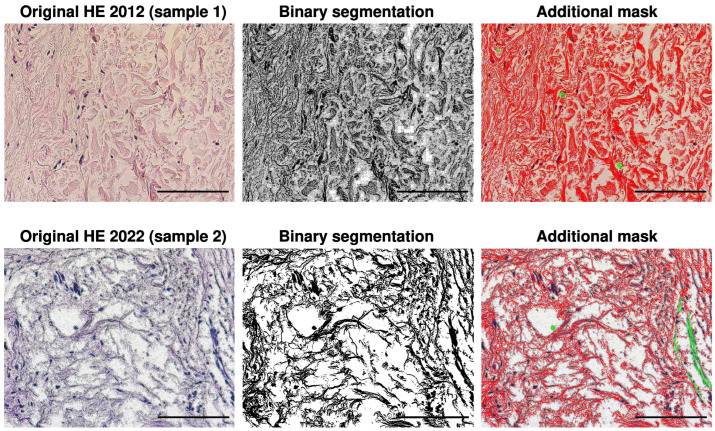
The original H&E, binary, and segmented images using color threshold from the volunteer in 2012 (**upper row**), and the original H&E, binary, and segmented images using color threshold from 2022 (**bottom row**). In the segmented images, fibers are marked in red, nuclei in blue, and artifacts or structures not considered in the analysis of red areas are additionally marked in green. The resolution of the images is 2572 × 1928 pixels with an objective magnification ×40 and scale bar 100 μm.

To acquire the images, a digital AxioCam color camera MRc5 with a CCD sensor resolution of 2584×1936 pixels (approximately 5 Mpix) and an Imager D1 microscope (Zeiss, Oberkochen, Germany) were utilized. The segmentation process was carried out using ImageJ-Fiji 1.54 software [31], with manual correction also being applied. When measuring the maximum fiber thickness using ImageJ’s Local Thickness plug-in, additional images were created with masks to mark the widest points of the thickest fibers (Figure 1).

### 2.4. Digital Analysis of Dermis

In image analysis, various descriptors are commonly used to identify object features. For skin features, indicators such as the area occupied by collagen and elastic fibers, as well as the maximum bundle width, are frequently utilized. Histograms for areas and widths are used instead of a single aggregate value, providing a more comprehensive analysis. The analysis was performed using Matlab R2020a software, in conjunction with the Image Processing Toolbox and Statistical Toolbox.

The first method of image analysis focused on the area of the ECM. The second method, used only for H&E, analyzed fiber bundle thickness. The results were subjected to statistical methods, with data expressed as mean and confidence interval. The analysis of H&E images was performed to assess total collagen. Using threshold images with red from H&E images (Figure 1), the areas and widths of collagen fibers were measured and histograms were generated. The third method involved analyzing the areas occupied by collagen and elastic fibers. This produced a histogram showing the frequency of occurrence of bundles of fibers of different sizes. For this method, the histogram was modeled [32] using the generalized Pareto distribution due to the long tails in the obtained histograms [33]. This method was chosen after analyzing the histogram shapes and rejecting other distributions, such as exponential and half-normal distributions in fit tests.

## 3. Results

### 3.1. Histological Staining Analysis

**Figure 2:**
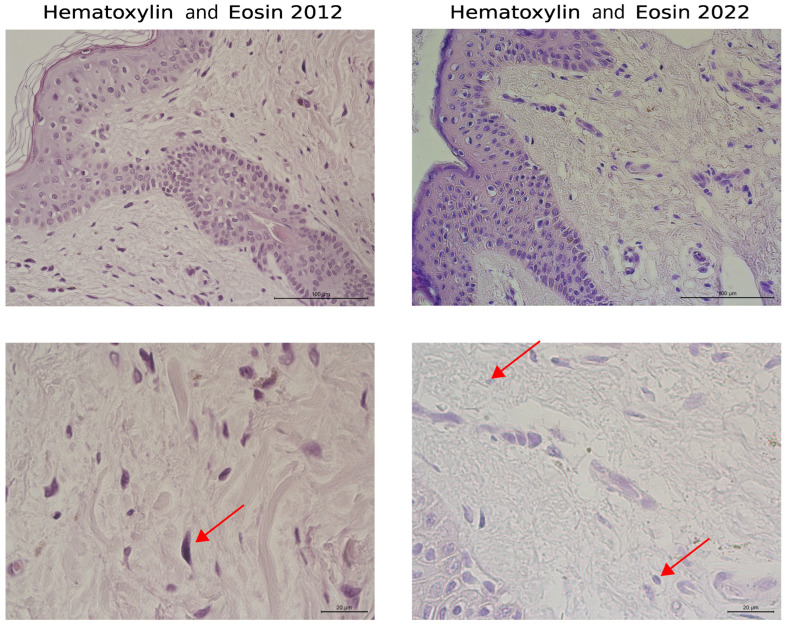
Skin dermis of the same volunteer at ages 47 years (2012) and 57 years (2022), stained with H&E. The upper panel images have an objective magnification of ×40, with a scale bar of 100 μm, while the lower panel images have an objective magnification of ×100, with a scale bar of 20 μm. Arrows indicate the dermal fibroblasts in the images.

**Figure 3:**
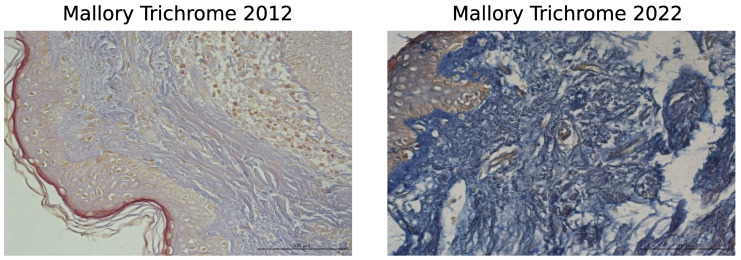

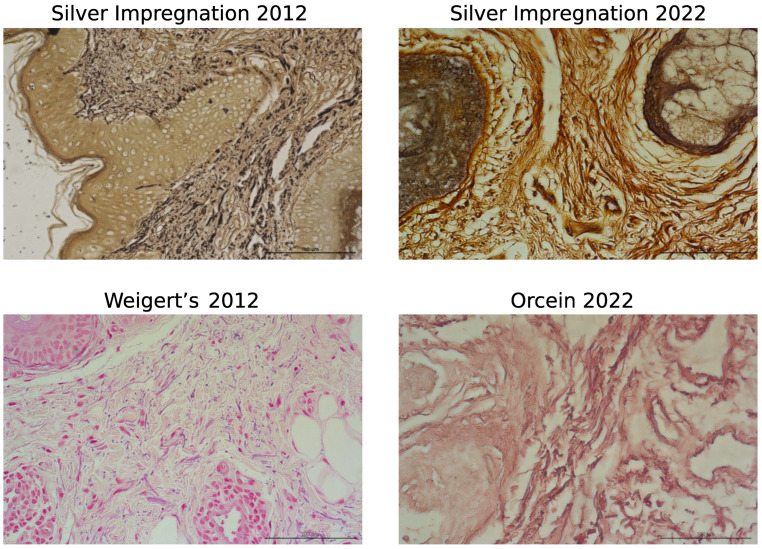
Skin dermis of the same volunteer at ages 47 years (2012) and 57 years (2022), stained with Mallory’s trichrome, silver impregnation, and Weigert’s/orcein. Objective magnification is ×40, and the scale bar represents 100 μm.

A comparison of the same volunteer’s dermal morphologies over 10 years (2012–2022) revealed significant changes in the non-cellular elements of the dermis, including type I and III collagens and elastic fibers. In 2012, collagen fibers in the papillary dermis were very thin, forming a loose network, while in the reticular layer, collagen fibers were tightly packed into bundles. Ten years later, the collagen fibers had become much thinner and fragmented in both the papillary and the reticular dermis. Additionally, the general structure of the bundles and collagen fibers was disrupted due to their dispersion (Figure 2, upper and lower panels). The shape of fibroblasts also changed with age, shifting from elongated to oval. Elongated fibroblasts were observed between collagen fiber bundles in younger skin, whereas oval fibroblasts predominated in the dermis 10 years later (Figure 2, lower panel). Changes in the organization of type I collagen were noted in Mallory’s trichrome-stained images, as well as in type III collagen (silver impregnation) and elastic fibers (Figure 3).

### 3.2. Digital Analysis of Dermis

#### 3.2.1. Analysis of the Area Occupied by the ECM

The first step involved assessing the area occupied by the ECM in the entire image of the dermis from slides taken in 2012 and 2022. These slides were stained using various methods, including H&E, Mallory’s trichrome, silver impregnation, and Weigert’s/orcein (Table 1).

#### 3.2.2. Collagen Type I Bundle Content in H&E Analysis

**Figure 4:**
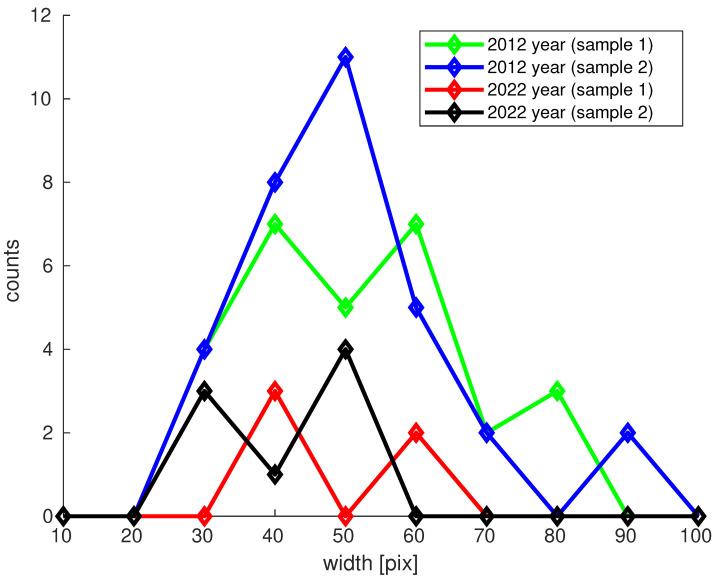
Histogram of the width of collagen fiber bundles observed in H&E images.

The second step involved analyzing H&E images to assess the content of collagen type I bundles. The percentage of the area occupied by collagen fibers (stained red) was estimated from the images (Figure 3). Mean and confidence interval (CI) values are provided in Table 2, with a histogram presented in Figure 4.

Visible changes were observed in the thickness of collagen fiber bundles in the ECM of the dermis between 2012 and 2022. The average width of the bundles was 51.1% in younger skin and 43.7% in older skin, indicating a mean width reduction of 7.4%. However, there was no significant difference between the distributions (CI 95%).

#### 3.2.3. Generalized Pareto Distribution (GPD) Analysis of the Area of ECM Fibrous Components

**Figure 5:**
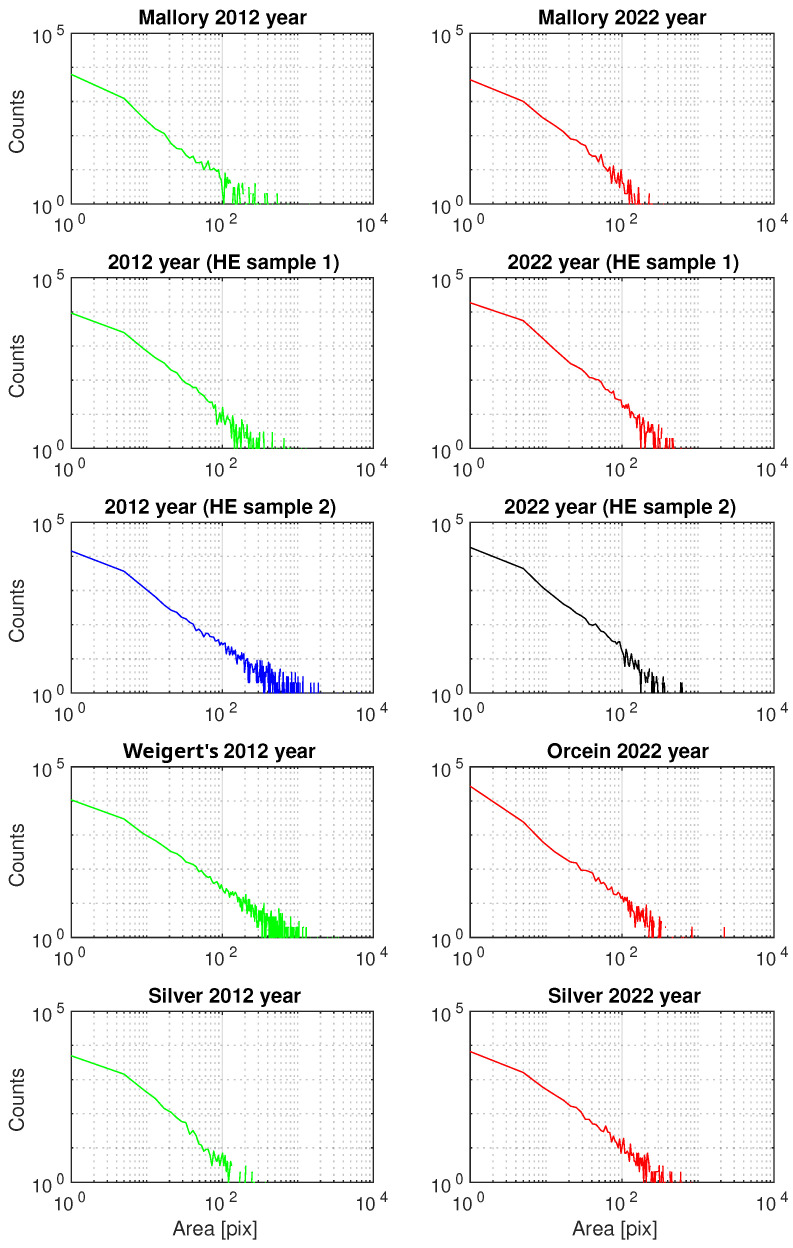
Histograms of segmented images stained with Mallory’s trichrome, H&E, and Weigert’s/orcein, representing collagen type I (H&E, Mallory), elastic fibers (Weigert’s/orcein), and collagen type III (silver). Green and blue—2012, red and black—2022.

**Figure 6:**
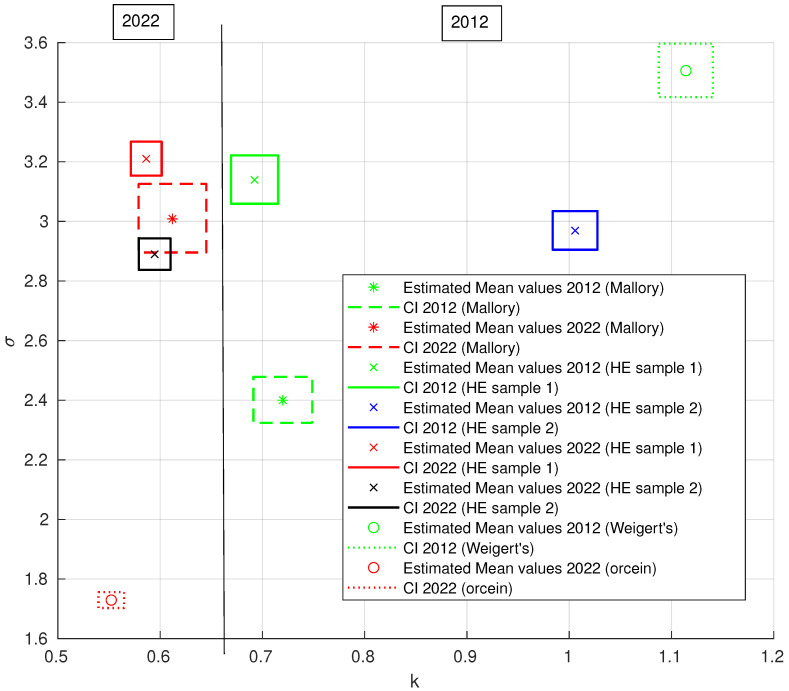
Estimated *k* and σ values for two-parameter generalized Pareto distribution (GPD) with 95% confidence intervals (rectangle) for Mallory’s trichrome, H&E, and Weigert’s/orcein-stained images. Green and blue—2012, red and black—2022.

The third step involved modeling a histogram using the generalized Pareto distribution (GPD) with two parameters: (*k*-tail/shape) index parameter and σ scale parameter. This was performed using the Matlab function ’gpfit’ to visualize the differences between the 2012 and 2022 samples for H&E, Mallory’s trichrome, silver, and orcein staining. Histograms of the fibrous areas of the ECM components are shown in Figure 5, and the estimated results are presented in Table 3. The grouping of the *k* and σ parameter results is illustrated in Figure 6.

The vertical line at k=0.66 indicates the distribution of results between 2012 and 2022, with *k* serving as a discriminating parameter. A *k* threshold of approximately 0.66 was identified, where larger values of *k* correspond to a higher frequency of fiber bundles with large surface areas, and smaller values correspond to a higher frequency of fiber bundles with smaller areas. Variability in individual parameters was observed for type I and type III collagen and for elastic fibers. This variability was evident both between different types of staining in the same year and, importantly, between the images from 2012 and 2022.

The GPD is often used to model the tails of other distributions and is particularly useful for long-tailed data, providing a flexible framework for estimating tail probabilities. In the case of GPD estimation with two parameters, class separation was achieved using the *k* parameter, although both *k* and σ parameters affect the distribution shape. Since the GPD does not ignore the long tail, it helps to find the relationship between the tail and the left side of the distribution. This suggests that the presence of extreme cases, such as fiber bundles with large surface areas, may be an important indicator of changes.

**Figure 7:**
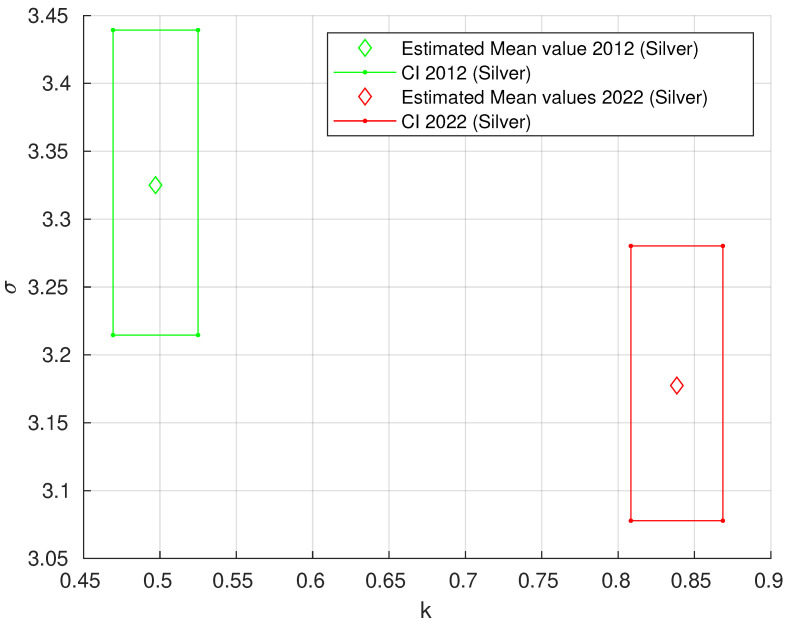
Estimated *k* and σ values for the two-parameter generalized Pareto distribution (GPD) with 95% confidence intervals (rectangle) for silver impregnation. Green—2012, red—2022.

The results for Mallory’s trichrome, H&E, and orcein staining are presented together because they exhibited a similar trend. As shown in Table 3, the grouping of k and σ results is visualized in Figure 6, and the vertical line shows the distribution of results between 2012 and 2022, with *k* as the main parameter. The grouping of *k* and σ values for silver impregnation is illustrated in Figure 7.

## 4. Discussion

According to previous research, the aging of fibroblasts, the main component of the dermis, increases the degradation of extracellular matrix elements [34]. In our study, we analyzed the morphological changes in the dermis of a man by examining his skin twice, with a 10-year interval between examinations. The skin samples were obtained from the postauricular area, which had limited sun exposure, thus mainly reflecting biological aging due to internal factors. This study examined the fibrous components of the extracellular matrix, including type I and type III collagen, which constitute most of the collagen in human skin, as well as the morphology of the elastic fibers of the dermis.

With age, the number of fibroblasts in the dermis decreases, affecting the amount and organization of collagen and elastic fibers [35]. Comparing the organizations of collagen bundles in the dermis at the two time points, we identified aging-related changes such as collagen bundle thinning, fragmentation, and the presence of wide spaces between them in the ECM. These features were more pronounced in the older dermis.

A study by another group analyzed the sun-protected skins of volunteers aged 25 ± 5 years and 75 ± 6 years, revealing similar characteristics of collagen in the dermis of older participants [36]. They noted that chronological aging resulted in biological changes to collagen fibers in the dermis, leading to increased stiffness and hardness, as well as a more reticular layer structure compared with the papillary layer. These changes in arrangement may be due to spaces created by the fragmentation of collagen fibers [18,37]. Furthermore, alterations in the structural and quantitative organization of the ECM in the dermis during aging are mainly due to a decrease in the proliferative and secretory activity of skin fibroblasts [38]. This degradation of the ECM causes an alteration of mechanical forces and disrupts interactions between fibroblasts and collagen fibers and other ECM elements, generating an aged fibroblast phenotype with changed cell shapes [18]. In our study, we noted a change in the shape of fibroblasts from elongated to oval over the 10-year period, indicating aging.

Quantitative digital analysis of the dermis provided more detailed results than morphological evaluation. However, using a simple estimator, such as the area for the whole image, did not yield the expected distinction between the two dermis samples studied. This parameter includes various components of the extracellular matrix, such as amorphous basic substances, collagens, and elastic fibers. In this study, the modification of collagen resulted in significant differences in the area occupied by the ECM in the skin sections from 2012 and 2022. As noted in morphological examinations, collagen degradation resulted in a significant dispersion of collagen and the presence of open spaces between fibers. Additionally, there were differences in the width of collagen bundles between the younger and older dermis, with confidence intervals near 100%.

Morphological and digital methods, including GPD, were used to evaluate changes in the ECM during the aging process. In our study, GPD analysis showed that, at the 2012 time point, there were more large bundles than small ones, whereas in 2022, the opposite was observed. The width of the bundles decreased by 7.4% in the dermis over the 10-year interval. These observations align with the structural changes in collagen organization commonly associated with skin aging [21]. These results may reflect decreased type I collagen synthesis and increased degradation processes.

According to GPD, it appears that the surface area of type III collagen in the ECM of older dermis has increased, which may be related to the change in the type I/III collagen ratio. Type III collagen plays a supportive role in organizing and maintaining the extracellular matrix in the dermis, working in tandem with type I collagen. Maintaining the functional and structural properties of various organs requires a balanced ratio of type I to type III collagen. An increase in this ratio may indicate cardiovascular dysfunction and can also serve as an indicator of skin wound healing [39,40,41]. Our findings correspond with other studies, noting that the ratio of these two types of collagens in the skin changes with age, with the ratio of type III collagen to type I collagen increasing [42].

Elastin in elastic fibers is one of the longest-lived proteins in vertebrate tissues. Due to their low turnover, these fibers accumulate alterations over time from various influences [43,44]. Our study did not find any evidence of elastic fiber degradation, but we did observe a reduced area of the ECM with fibers in the GPD results. The skin evaluated was from an area with limited exposure to environmental factors.

It is postulated that the skin ages chronologically, and elastic fibers undergo structural changes. Intrinsic aging primarily involves the degradation of oxytalan fibers, specifically fibrillin-rich microfibrils [21]. As the dermis ages, the elastic fiber network undergoes various types of changes, such as mechanical fatigue, chemical modifications, and enzymatic degradation, leading to a gradual loss of skin elasticity [45]. Skin aging affects both sexes and is characterized by a gradual loss of skin elasticity, the appearance of fine wrinkles that deepen with age, and increased stiffness of the dermis [34].

With the development of aesthetic medicine, various methods are being sought to help improve the condition of aging skin in both women and men. Treatments are aimed at stimulating the proliferative and metabolic activities of dermal fibroblasts. One such method is the application of autologous fibroblasts isolated from the dermis and their injection after in vitro culture. Our earlier study showed that the number of dermal fibroblasts and the width of collagen fibers increased after cell injection [30]. Additionally, our research indicated that the optimal population of cultured in vitro fibroblasts with the best potential for synthesizing ECM elements is the second passage of the cells [46]. Further analysis of the autologous fibroblast transcriptome profile indicated that third-passage cells were strongly linked to the cell cycle and cell proliferation. Therefore, cells from both passages can be used for skin reimplantation and healing, with no expression of genes associated with the aging process [47].

The main limitations of this study include its reliance on material obtained from a single patient. However, it is important to note that these results are preliminary, and further studies with a larger number of volunteers of similar age are planned. The question of whether the observed changes are sudden or gradual cannot be answered based on just two measurements (2012 and 2022), which is a limitation derived from the sampling theorem. To determine this, it would be necessary to measure the same area of skin annually, but such data are currently not available.

Additionally, molecular analyses could be included to assess the up- and downregulation of genes encoding proteins involved in skin aging. Studies of male skin aging typically rely on measurements of linear facial photographs and non-invasive methods to compare individuals in different age groups [48]. However, this study is unique in that it compares samples from the same subject at two different and consecutive time points, allowing for the observation of morphological differences over a 10-year period.

## 5. Conclusions

This study presents, for the first time, a comparison of changes in the fibrous material of the ECM of the male dermis in the same volunteer over a 10-year interval. The obtained results shed light on the complex dynamics of skin aging, highlighting the key role of ECM elements such as collagen and elastic fibers in maintaining skin integrity. Through meticulous histological staining and morphological analyses, this study provides valuable insights into the morphological changes occurring in these fibrous structures over a decade.

While it is true that skin aging may occur at a slower rate in men than in women, especially on the face, it is important to recognize that modern aesthetic medicine and cosmetology also offer opportunities for men to address these concerns. Expanding research on male skin aging would help better understand the unique needs and concerns of men in this area. A further study of the mechanisms of skin aging in larger groups of patients is needed to develop therapeutic strategies to counteract the negative effects of aging on skin structure and function. This research could pave the way for improved skin care practices and ultimately enhance patient quality of life.

## Figures and Tables

**Table 1:** Mean and 95% confidence intervals (CIs) for measurements of the area occupied by the extracellular matrix (ECM) on slides stained with different methods.

Staining Procedure	Case	Mean [%]	Mean CI [%]	Statistical Difference
H&E	2012 sample 1	57.3	(56.4; 58.1)	Yes, but 2022
	2012 sample 2	40.7	(40.6; 40.7)	samples are
	2022 sample 1	54.7	(54.1; 55.2)	between 2012
	2022 sample 2	54.3	(53.7; 54.9)	samples
Mallory	2012	60.8	(59.7; 61.9)	yes
trichrome	2022	79.2	(77.3; 81.1)	
Silver	2012	78.7	(76.9; 80.3)	yes
impregnation	2022	59.5	(58.8; 60.6)	
Weigert’s	2012	48.6	(48.5; 48.8)	yes
Orcein	2022	52.6	(52.0; 53.1)	

**Table 2:** Number of cases, mean, and 95% confidence intervals (CIs) for the number of collagen fiber bundles and the maximum bundle width observed in H&E images.

	No. of Collagen Bundles	Mean [%]	Mean CI [%]	Max. Width of Bundle [pix]
2012	60 (sample 1 + sample 2)	51.1	(47.0; 55.1)	91.1
2022	13 (sample 1 + sample 2)	43.7	(38.1; 49.3)	61.1

**Table 3:** Estimated GPD parameters for H&E, Mallory’s trichrome, Weigert’s/orcein, and silver-stained images (95% confidence intervals).

Staining Procedure	Case	*k*	*k* CI	σ	σ CI
H&E	2012 sample 1	0.6923	(0.6693; 0.7154)	3.1394	(3.0592; 3.2216)
	2012 sample 2	1.0058	(0.9839; 1.0277)	2.9690	(2.9049; 3.0346)
	2022 sample 1	0.5864	(0.5713; 0.6015)	3.2099	(3.1535; 3.2674)
	2022 sample 2	0.5947	(0.5792; 0.6102)	2.8896	(2.8373; 2.9428)
Mallory	2012	0.7199	(0.6912; 0.7487)	2.4000	(2.3238; 2.4787)
trichrome	2022	0.6121	(0.5790; 0.6452)	3.0085	(2.8956; 3.1258)
Silver	2012	0.4971	(0.4693; 0.5249)	3.3251	(3.2146; 3.4393)
impregnation	2022	0.8385	(0.8083; 0.8686)	3.1774	(3.0778; 3.2803)
Weigert’s	2012	1.1141	(1.0876; 1.1406)	3.5058	(3.4173; 3.5966)
Orcein	2022	0.5522	(0.5397; 0.5647)	1.7294	(1.7029; 1.7564)

## Data Availability

The data presented in this study are available on request from the corresponding author.

## Abbreviations

The following abbreviations are used in this manuscript:

CCD	charge-coupled device
CI	confidence interval
ECM	extracellular matrix
GPD	generalized Pareto distribution
H&E	hematoxylin and eosin
MMPs	matrix metalloproteinases
